# An updated version of NPIDB includes new classifications of DNA–protein complexes and their families

**DOI:** 10.1093/nar/gkv1339

**Published:** 2015-12-09

**Authors:** Olga Zanegina, Dmitriy Kirsanov, Eugene Baulin, Anna Karyagina, Andrei Alexeevski, Sergey Spirin

**Affiliations:** 1 Belozersky Institute of Physico-Chemical Biology, Lomonosov Moscow State University, Moscow 119992, Russia; 2 OOO Lexpro Soft, Moscow 119019, Russia; 3 Laboratory of Applied Mathematics, Institute of Mathematical Problems in Biology, Puschino 142290, Russia; 4 Laboratory of Biologically Active Nanostructures, Gamaleya Center of Epidemiology and Microbiology, Moscow 123098, Russia; 5 Laboratory of Genome Analysis, Institute of Agricultural Biotechnology, Moscow 127550, Russia; 6 Sector of Applied Informatics, Research Institute for System Studies, Moscow 117218, Russia

## Abstract

The recent upgrade of nucleic acid–protein interaction database (NPIDB, http://npidb.belozersky.msu.ru/) includes a newly elaborated classification of complexes of protein domains with double-stranded DNA and a classification of families of related complexes. Our classifications are based on contacting structural elements of both DNA: the major groove, the minor groove and the backbone; and protein: helices, beta-strands and unstructured segments. We took into account both hydrogen bonds and hydrophobic interaction. The analyzed material contains 1942 structures of protein domains from 748 PDB entries. We have identified 97 interaction modes of individual protein domain–DNA complexes and 17 DNA–protein interaction classes of protein domain families. We analyzed the sources of diversity of DNA–protein interaction modes in different complexes of one protein domain family. The observed interaction mode is sometimes influenced by artifacts of crystallization or diversity in secondary structure assignment. The interaction classes of domain families are more stable and thus possess more biological sense than a classification of single complexes. Integration of the classification into NPIDB allows the user to browse the database according to the interacting structural elements of DNA and protein molecules. For each family, we present average DNA shape parameters in contact zones with domains of the family.

## INTRODUCTION

Currently about 3000 3D structures of DNA–protein complexes are known. Variety of DNA–protein interactions can be described in quite different terms attributed to, for example, specific and nonspecific recognition; interaction with the major or the minor DNA grooves; interaction via alpha-helices, beta-structures or unstructured regions of protein; interactions of different kinds: hydrogen bonds, water-mediated contacts or hydrophobic interaction; etc. An adequate description of the ways DNA and protein interact with each other, as well as a systematization of DNA–protein complexes are important for understanding fundamental mechanisms of intermolecular interaction.

The database NPIDB (1), http://npidb.belozersky.msu.ru/, contains structural information about all DNA–protein and RNA-protein complexes available from PDB (2). In this work, we describe an updated version of NPIDB, which includes a new classification of complexes of double-stranded DNA with proteins.

Several authors proposed structural classifications of DNA–protein complexes. A structural taxonomy of DNA-binding protein domains was described in Harrison *et al*. (3) and then refined in Luisi *et al*. (4). Wintjens with co-authors (5) developed the procedure of automatic classification of short protein fragments and applied it to the classification of helix-turn-helix DNA-binding domains (6). Luscombe with co-authors (7) divided 240 DNA–protein complexes into 8 functional groups and further into 54 structural families on the basis of the structural similarity of DNA-binding motifs of proteins. Malhotra and Sowdhamini (8) further evolve this classification: the selected families were expanded and new groups and families were added. Ponomarenko with co-authors (9) proposed a fully automatic classification of 338 protein SCOP domains from 269 DNA-binding proteins based on structural similarities of DNA-binding protein patterns. Prabakaran with co-authors (10) divided 62 DNA–protein complexes into seven clusters using automatic classification based on 11 descriptors, in particular: the number of atomic bonds between protein and DNA in the major or the minor grooves, depth of the DNA grooves, DNA bending, GC-content of the DNA and the area of the DNA–protein contact. It was shown that the similarity of structures of DNA-binding motifs does not necessarily provide the similarity of modes of DNA–protein recognition. In this regard, the authors emphasize that not only the properties of a DNA-binding protein but some general parameters of interaction should be considered in classification procedure. The authors use descriptors characterizing primarily DNA but not protein; thus this classification can be considered as DNA-based. Another DNA-based classification was proposed in the paper of Sathyapriya with co-authors (11). The graphs of DNA–protein interactions for 118 DNA–protein complexes were built, and the DNA–protein complexes were divided into seven classes depending on the parts of the DNA that contact amino acid residues of the protein. As in the previous work, in the proposed classification the same protein motifs (for example, the helix-turn-helix motif) appeared in various classes, and each class included different motifs. Biswas with co-authors (12) considered and classified DNA–protein contacts based on the secondary structure protein elements exposed on DNA–protein interface. In some cases, an obvious contradiction was observed between the SCOP (13,14) class of protein (α, β, α/β, α+β and others) and interface class (α, β, αβ, nonregular) defined in (12). The interface-based classes were also used for defining of functional groups in the work of Luscombe with co-authors (7). Schneider with co-authors (15) analyzed DNA–protein complexes on the basis of classification of interacting protein blocks of five consecutive amino acid residues, on the one hand, and structures of dinucleotide conformers, on the other hand. As a result the frequency of contacts for different structural elements of different protein classes, such as transcription factors, nucleases, *etc*. was calculated.

In our classification, we do not consider entire structures of protein–DNA complexes but extract DNA-binding protein domains complexed with DNA. Taking into account that structural similarity of DNA-binding domains does not necessarily results in similar way of DNA recognition (8), we use both DNA and protein features in our classification. The analyzed material included 1975 structures of protein domains from 905 biounits described in 756 PDB entries, which is several times greater than in the previously proposed classifications. Unlike previously proposed classifications, we classified not only individual complexes of protein domains with DNA, but also the families of related complexes. In addition, we took into account not only hydrogen bonds, but also hydrophobic interaction between macromolecules. We have identified 99 interaction modes of individual protein domains with DNA and 17 interaction classes of DNA-binding domain families. We have developed and described a pipeline to add new complexes and families into the developed classification. The classification results are integrated into NPIDB. Also for the classified families of DNA-binding domains we present information on a number of DNA shape parameters in contact zones of domains of the families. Our classification may simplify navigation through numerous structures of DNA–protein complexes considering peculiarities of DNA–protein interaction.

## MATERIALS AND METHODS

In this paper a *protein domain* is a structural protein domain according to SCOP.

We considered complexes of protein domains with the double DNA helix of 10 or more bp. Single nucleotide loops or nucleobase excisions within the contact area were allowed. DNA helices were detected by the program 3DNA (16).

We considered two kinds of contacts between protein and DNA molecules: hydrogen bonds between protein and DNA atoms and hydrophobic clusters that include protein and DNA atoms simultaneously. Ion (electrostatic) interactions are considered as particular cases of hydrogen bonds. Water-mediated contacts are not considered because in the used set of structures there is some amount of low-resolution structures, which do not contain water molecules. Hydrogen bonds are detected according to the protocol described in the help page of NPIDB (http://npidb.belozersky.msu.ru/help.html?div=interaction). In brief, two atoms of nitrogen or oxygen, one from DNA and one from protein, form a hydrogen bond if: (i) their centers are closer than 3.7 Å from each other; (ii) their ‘hydrogen bonding power’ is >0.1. The hydrogen bonding power is a conditional value depending on the configuration of the atoms and their covalent bonded neighbors (see the formulas at the NPIDB help page). A hydrophobic contact is detected between two non-polar atoms, one from DNA and one from protein, if: (i) the distance between their centers is less than 5.4 Å; (ii) they are not separated by other atoms, i.e. the straight line segment connecting their centers does not intersect van der Waals spheres of other atoms; (iii) they belong to the same hydrophobic cluster. Hydrophobic clusters were determined by the program CluD (17). In this work, we used the following list of non-polar atoms: all carbon and sulphur atoms of protein that are not covalently bonded with oxygen or nitrogen atoms, and all carbon atoms of DNA.

Three main notions that are used in this paper are: **contact type** of a particular DNA–protein contact, **interaction mode** of a structure of protein domain–DNA complex, and **interaction class** of a family of DNA-binding protein domains. For each DNA–protein contact we define its *type* (*contact type*) as the pair of interacting elements (one of protein and one of DNA), to which the contacting atoms belong.

Interacting elements of protein are secondary structure elements: helix (alpha-helix or 3_10_-helix), beta-strand or loop (turn or unstructured segment). Secondary structure was determined by the program Stride (18). Interacting elements of DNA are the sugar-phosphate backbone, the DNA major groove, and the DNA minor groove. In this work, only contacts of nucleobase (not backbone) atoms are regarded as contacts of the DNA grooves. Backbone atoms are atoms of deoxyribose and the phosphate groups of DNA. Atoms of the major groove are C5, C6, N6, N7 and C8 of adenine, C4, O4, C5, C6, C7 of thymine, C5, C6, O6, N7 and C8 of guanine and C4, N4, C5, C6 of cytosine. Atoms of the minor groove are N1, C2, N3 and C4 of adenine, C2, O2 and N3 of thymine, N1, C2, N2, N3 and C4 of guanine and C2, O2 and N3 of cytosine.

In total, there are nine contact types (in parentheses are the designations that we use):

- contacts of protein domain with the sugar-phosphate backbone of DNA by: (a) helix (**H – Bb**), (b) beta-strand (**S – Bb**), (c) loop (**L – Bb**);
- contacts of protein domain with the DNA major groove by: (d) helix (**H – Mj**), (e) beta-strand (**S – Mj**), (f) loop (**L – Mj**);
- contacts of protein domain with the DNA minor groove by: (g) helix (**H – Mn**), (h) beta-strand (**S – Mn**), (i) loop (**L – Mn**).

**Figure 1.**
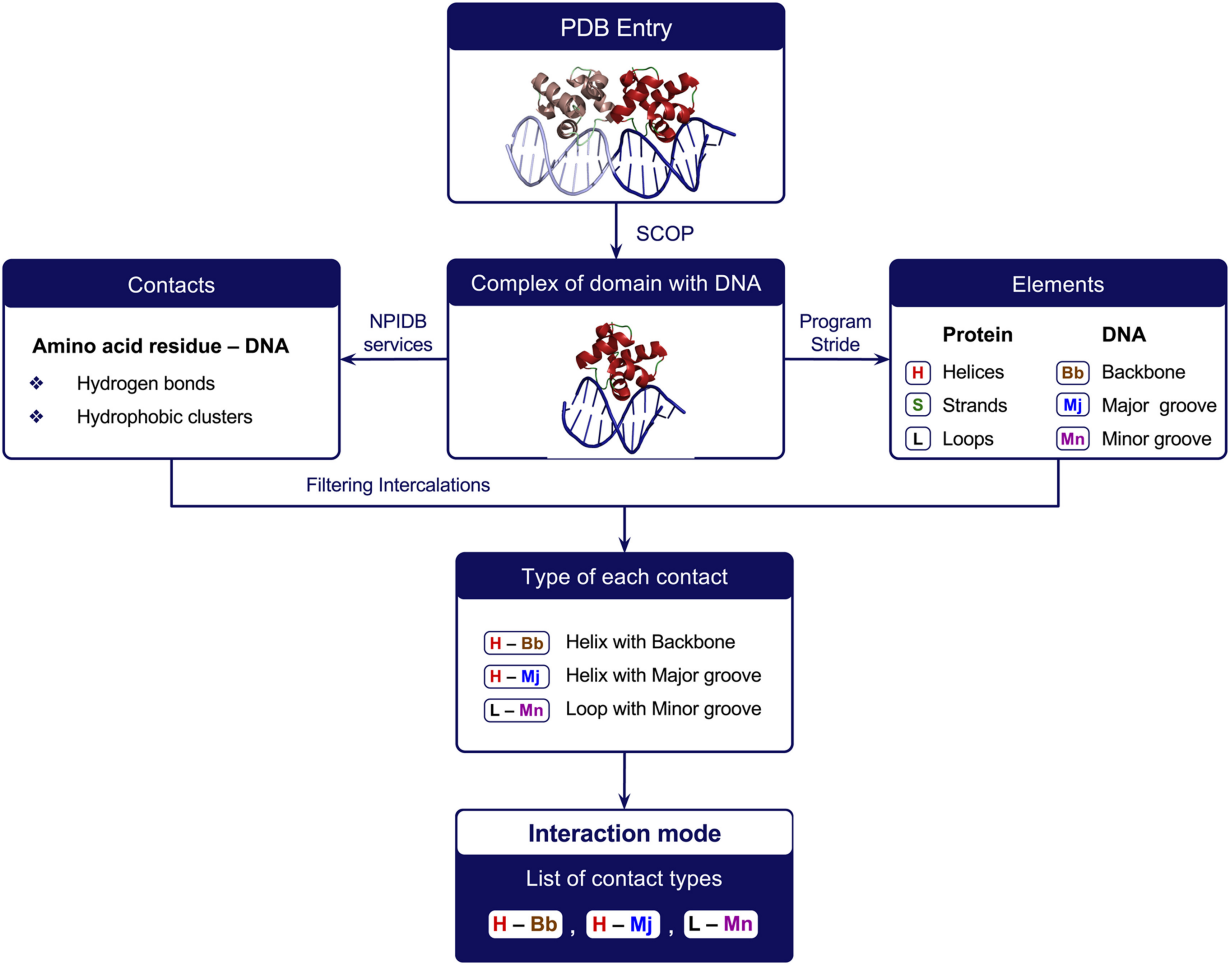
Workflow for determination of the interaction mode for a complex of a protein domain with DNA.

The *interaction mode* of a protein domain in a given DNA–protein complex is the list of contact types detected. For example, the record ‘(H – Mj) (S – Mn) (L – Bb) (L – Mn)’ indicates that the protein domain contacts with the DNA backbone by loop(s), with the DNA major groove by helix(es) and with the DNA minor groove by beta-strand(s) and loop(s). See Figure 1 for the workflow of determination of interaction modes.

Each protein domain in complex with DNA can be represented in PDB several times, in one or more PDB entries. Interaction modes of protein domains can vary significantly even for different structures of the same DNA–protein complex. If in at least one structure some contact type is detected, then such contact type is considered as possible for this domain in general.

A *family* of DNA-recognizing protein domains is a family according to SCOP in most cases. The only exception is the ‘Leucine zipper’ family that was divided into two subfamilies (Leucine zipper I and Leucine zipper II) because domains of these subfamilies interact with DNA in quite different manner.

A contact type is *characteristic* for a family if it is detected in at least one structure of each domain of that family. In other words, a contact type is characteristic if for each domain of the family it belongs to the union of all contact types observed in all structures of this domain. The reason to use the union of contact types is as follows. If some contact type (for example, H-Mj, *e.g*., an interaction between an alpha-helix and the DNA major groove) is presented in one structure but is absent in other structures of the same domain, we interpret this situation as a principal possibility for this domain to interact this way with DNA. Thus the union of all contact types observed in structures of one particular domain contains all contact types that can be supposed as possible for this domain.

**Figure 2.**
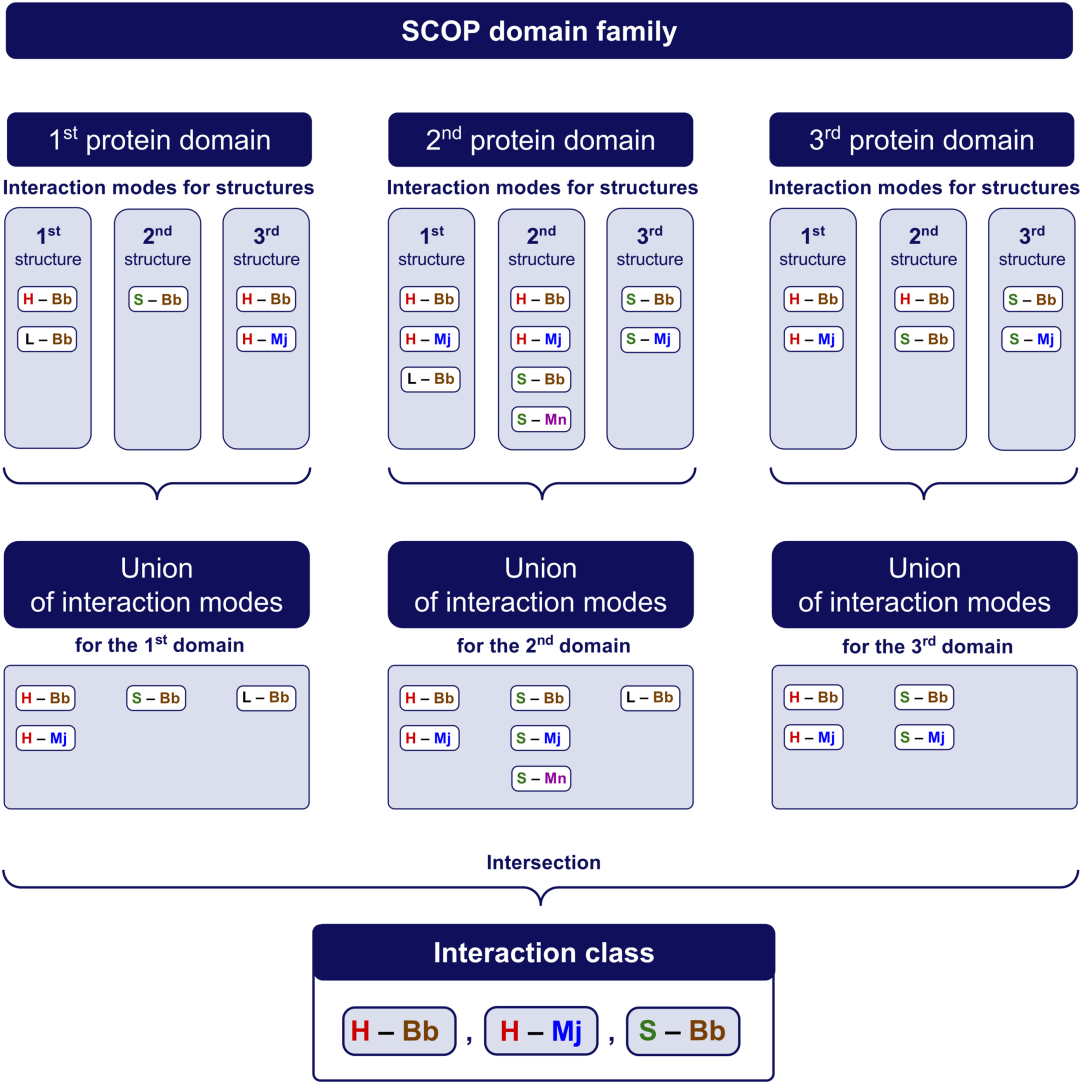
Determination of the interaction class of a family.

The *interaction class* for a family of DNA-recognizing protein domains is the list of all characteristic contact types of protein–DNA contacts for the family. In other words, the interaction class of a family is the intersection of unions of interaction modes, see Figure 2. If the interaction class defined as above is empty, i.e. there is no characteristic contact types, then we say that the interaction class of this family is *miscellaneous*.

748 DNA–protein complexes with double-stranded DNA of 10 or more bp were obtained from database NPIDB (1). From these complexes, 1942 structures of 314 DNA-binding protein domains were extracted. These domains represent 115 families.

A *contact zone* of a DNA-binding domain in a particular structure consists of this domain and all nucleotides of DNA that are either in contact with this domain or are within two nucleotides along the chain from some nucleotide that contacts the domain.

The program Curves+ (19) was used to compute shape parameters of DNA double helices. If a contact zone of some protein domain in some structure includes a double helix of at least three base pairs, then the following parameters were extracted from the Curves+ output: the mean value of Ax-bend, which is the angle between axes of the DNA helix in two adjacent base pairs, and the mean values of width and depth of two DNA grooves (if the program Curves+ detects the corresponding groove of the helix). For each domain, we computed the average of these five parameters for all structures of this domain. For each family, we computed the average, the minimum and the maximum of the shape parameters for all domains of the family.

## NEW DATABASE CONTENT AND WEB INTERFACE

### List of interaction modes for DNA–protein structures

Formally there are 511 possible interaction modes. Among the analyzed complexes totally 97 interaction modes were detected.

The list of interaction modes is available at http://npidb.belozersky.msu.ru/interactionmodes.html. It is organized as a table of three columns. The first column contains symbolic denotations of interaction modes (e.g. ‘(H–Bb) (H–Mj) (L–Bb)’ or ‘(L–Bb) (L–Mj) (L–Mn)’, see Materials and Methods). The second column contains numbers of protein domain families whose representatives interact with DNA by a certain mode. The third column contains numbers of DNA–protein domain structures with a certain interaction mode. The table is sorted according to the number of SCOP families including domains with the corresponding interaction modes.

Each interaction mode has its own page (e.g. http://npidb.belozersky.msu.ru/mode/interactionmodes.html?_H-Bb_L-Bb_) with the list of structures grouped into SCOP families.

### List of interaction classes for domain families

In total, there are 115 domain families presented in structures of complexes with the double-stranded DNA, including two subfamilies of ‘Leucine zipper’ family (see Materials and Methods). We classified 34 domain families that have three or more representatives. In the future, addition of new structural data will allow to classify more families as well as to improve the existing classification.

A contact type that is detected (at least in one structure) for each protein domain of a family is regarded as characteristic for the entire family. The interaction class of a family consists of characteristic contact types. As a result, 34 families are classified onto 17 interaction classes (Table 1). The list of interaction classes is available at http://npidb.belozersky.msu.ru/contacttypesnew.html. It is organized as a table of four columns. The first column contains symbolic denotations of interaction classes (e.g. ‘(H–Mj) (H–Bb) (L–Bb)’ or ‘(L–Bb) (L–Mj) (L–Mn)’, see Materials and Methods). The second column contains numbers of SCOP families in corresponding interaction classes. The third column contains numbers of structures of all domains belonging to these families. The fourth column contains numbers of different domains.

**Table 1.** Interaction classes of SCOP families with average values of DNA shape parameters

Interaction class	Families (SCOP ID)	Ax–bend	MnW	MnD	MjW	MjD
Miscellaneous	Middle domain of MutM-like DNA repair proteins (a.156.1.2)	0.2	–	–	–	–
	NF-kappa-B/REL/DORSAL transcription factors, C-terminal domain (b.1.18.1)	–	–	–	–	–
	Classic zinc finger, C2H2 (g.37.1.1)	0.7	7.5	4.4	11.3	2.0
	C-terminal, Zn-finger domain of MutM-like DNA repair proteins (g.39.1.8)	0.3	–	–	–	–
H – Bb	AraC type transcriptional activator (a.4.1.8)	0.7	–	–	–	–
	Replication initiation protein (a.4.5.10)	0.7	7.5	4.6	12.5	5.6
	Transcription factor IIB (TFIIB), core domain (a.74.1.2)	0.3	9.2	3.3	–	–
	Leucine zipper domain (h.1.3.1) I	1.0	11.9	1.5	–	–
L – Bb	DnaQ-like 3′-5′ exonuclease (c.55.3.5)	–	–	–	–	–
S – Bb L – Bb	N-terminal domain of MutM-like DNA repair proteins (b.113.1.1)	0.5	–	–	–	–
H – Bb S – Bb L – Bb	Nucleosome core histones (a.22.1.1)	1.0	6.1	4.9	11.0	4.6
H – Bb H – Mj	HLH, helix-loop-helix DNA-binding domain (a.38.1.1)	1.0	7.9	4.1	11.2	1.9
	Leucine zipper domain (h.1.3.1) II	0.7	7.2	4.5	13.1	2.1
H – Bb H – Mj L – Bb	POU-specific domain (a.35.1.1)	0.8	9.8	0.6	11.4	1.2
	Phage repressors (a.35.1.2)^a^	1.8	7.1	4.6	12.5	4.2
	Homeodomain^a^ (a.4.1.1)	1.1	5.9	4.8	12.5	4.5
	Myb/SANT domain (a.4.1.3)	0.5	6.4	4.5	–	–
	ets domain (a.4.5.21)	0.9	8.2	3.8	13.6	6.3
	Interferon regulatory factor (a.4.5.23)	1.6	5.8	5.2	11.0	5.1
	Nuclear receptor (g.39.1.2)	0.9	6.5	4.8	12.3	4.0
H – Bb H – Mj S – Bb	Viral DNA-binding domain (d.58.8.1)	2.8	7.1	4.6	8.4	5.2
H – Bb L – Bb L – Mj	Rel/Dorsal transcription factors, DNA-binding domain (b.2.5.3)^a^	1.2	5.0	5.3	12.1	4.4
H – Bb H – Mj L – Bb L – Mj	Zn2/Cys6 DNA-binding domain (g.38.1.1)	1.5	7.4	4.1	11.3	0.6
	Zinc finger design (k.12.1.1)	1.1	6.3	4.6	13.2	5.8
H – Bb S – Bb S – Mj L – Bb L – Mj	Group I mobile intron endonuclease (d.95.2.1)	1.8	6.2	4.9	13.9	4.2
H – Bb H – Mn L – Bb	HMG-box^a^ (a.21.1.1)	5.1	10.9	1.2	10.6	4.8
H – Bb S – Bb L – Bb L – Mn	Prokaryotic DNA-bending protein (a.55.1.1)	2.4	11.0	1.1	11.3	-1.2
H – Bb H – Mj L – Bb L – Mn	Recombinase DNA-binding domain (a.4.1.2)	1.7	5.5	5.5	11.2	3.6
	Paired domain (a.4.1.5)	1.2	7.8	4.7	9.7	5.7
	SRF-like (d.88.1.1)	2.7	6.7	5.1	10.9	4.1
	Lambda integrase-like, catalytic core (d.163.1.1)	1.6	6.0	4.7	12.2	5.3
S – Bb S – Mn L – Bb L – Mn	TATA-box binding protein (TBP), C-terminal domain (d.129.1.1)	3.6	11.9	-0.5	–	–
H – Bb H – Mj H – Mn L – Bb L – Mj	GalR/LacI-like bacterial regulator (a.35.1.5)	3.4	8.4	4.3	12.7	3.4
H – Bb L – Bb	Restriction endonuclease FokI, N-terminal (recognition) domain^a^ (a.4.5.12)	1.1	–	–	12.6	-0.4

^a^families for which new structures will be likely to improve the classification by enlarging the list of contact types.

In the column ‘Interaction class’ H is for helix, S is for sheet, L is for loop or unstructured segment of protein, Bb is for the DNA backbone, Mj is for the DNA major groove, Mn is for the DNA minor groove. Right five columns contain the average values of: Ax-bend, that is the mean angle between axes of the DNA helix in subsequent base pairs in contact zone of a domain, the width of the DNA minor groove (MnW), the depth of the DNA minor groove (MnD), the width of the DNA major groove (MjW), and the depth of the DNA major groove (MjD). Ax-bend is in degrees, the widths and depths are in Angstroms. The dash (‘–’) means that this parameter cannot be computed for structures of the family.

Each interaction class has its own web page (*e.g*., http://npidb.belozersky.msu.ru/families/contacttypesnew.html?_H-Bb_H-Mj_L-Bb_L-Mn_) with a table of families with this interaction class. The table contains information on number of structures, number of different domains and interaction modes realized in different structures. Also we included the information on DNA shape in contact zones of domains of each family involved into the classification. Namely, for each of the parameters Ax-bend, width and depth of the minor groove and width and depth of the major groove, the table contains its average value, maximum and minimum for domains of the family. For some families, some parameters may be computed only for one domain, in these cases only one value are presented. Also for some families some parameters may be not computed even for one structure, in this case the corresponding cell of the table contains the dash ‘–’. For example, if in all structures of the family the DNA in contact zones do not contain at least three base pairs, then all five parameters are not computed (this is the case of two families, b.1.18.1 and c.55.3.5). The DNA major and minor grooves are not determined by the program Curves+ in many cases, that is why the dashes occur in the corresponding columns rather often.

### Example of application

Suppose we would like to find structural families that include many domains able to interact with DNA in a manner close to that of homeodomains. From the page of homeodomain SCOP family (a.4.1.1) we go to the page of its interaction class: http://npidb.belozersky.msu.ru/families/contacttypesnew.html?_H-Bb_H-Mj_L-Bb_. From the table of families with this class we can see that there are five interaction modes detected for different structures of the homeodomain family. The page of each mode is available through the corresponding hyperlink from the last column of the table. Visiting each page we can obtain lists of families with each mode. Only two interaction modes, namely, ‘(H – Bb) (H – Mj) (L – Bb)’ and ‘(H – Bb) (H – Mj) (L – Bb) (L – Mn)’, are represented by a large number of structures (17 and 33 structures, correspondingly). Among families with representatives of the first mode are ‘lambda integrase-like, N-terminal domain’ (a.60.9.1, 48 structures), ‘CAP C-terminal domain-like’ (a.4.5.4, 15 structures) and ‘GerE-like (LuxR/UhpA family of transcriptional regulators)’ (a.4.6.2, 15 structures). The second mode is detected for 23 structures of complexes of domains of the family ‘Lambda integrase-like, catalytic core’ (d.163.1.1). From 1 to 11 structures of both modes are presented in other families. So among 115 SCOP families we have selected four families with high amount of representatives, which is enough for a comparative analysis.

## ANALYSIS OF CLASSIFICATION RESULTS

### Distribution of available DNA–protein complexes among interaction modes

Interaction modes are very different in numbers of structures and families where they are detected. For example, 11 interaction modes are detected in ten or more families. At the same time, there are 33 interaction modes detected only in one family each and 23 modes even in one structure each. Some of the rare modes may be results of artifacts of X-ray structures or secondary structure detection.

The same interaction mode can be observed for domains of different SCOP folds and even classes. For instance, the contact of protein helices, beta-strands and loops with the DNA backbone and major groove ‘(H – Bb) (S – Bb) (S – Mj) (L – Bb) (L – Mj)’ is observed in families ‘Trafficking protein A-like’, ‘Rel/Dorsal transcription factors(DNA-binding domain)’, ‘Restriction endonuclease EcoRV’, ‘Group I mobile intron endonuclease’ and ‘Eukaryotic DNA topoisomerase I (N-terminal DNA-binding fragment)’ belonging to the classes α, β, α/β, α+β and multidomain proteins, respectively. Even for domains of the class α, beta-strands may play a significant role in DNA recognizing, despite they occupy only a small part of the domain structure.

### Variation of interaction mode within one family

In most cases the mutual orientation of DNA and protein domain is similar for DNA–protein complexes of different protein domains from the same family. Nevertheless sometimes protein domains from one family interact with DNA in different manner.

**Figure 3.**
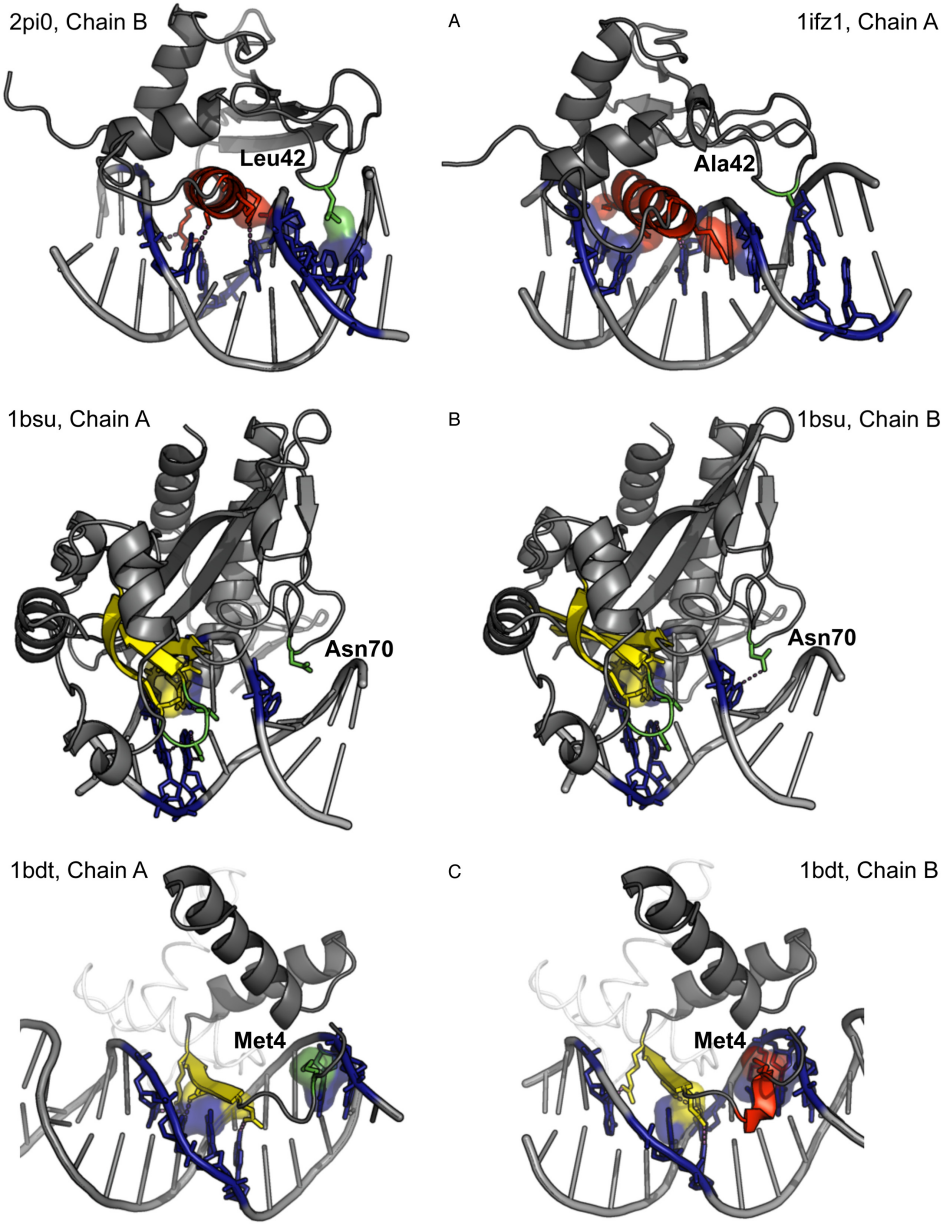
Variations of DNA interaction modes within one SCOP family. Only contacts with DNA grooves are shown. Hydrophobic clusters are represented with their surfaces, hydrogen bonds with dashed lines. (**A**) Family ‘Interferon regulatory factor’, an additional contact due to an aa residue replacement; (**B**) family ‘Restriction endonuclease EcoRV’, the presence of a contact depends on conformation of a side chain; (**С**) family ‘Arc/Mnt-like phage repressors’, the type of the contact, L – Mj or H – Mj, depends on detection of 3_10_-helix (in red) or unstructured segment (in green) by the program Stride.

Variations in interaction mode can be due to different length, composition or mobility of unstructured segments. For example, in the family ‘Interferon regulatory factor’ in some structures (*e.g*., PDB code 2PI0) there is a hydrophobic contact of a loop with the DNA minor groove. This contact is observed only for proteins that contain Leu-42 (Figure 3a, left). For these structures, the interaction mode is ‘(H – Bb) (H – Mj) (S – Bb) (L – Bb) (L – Mn)’. For the members of the family that contain Ala at the same position, the interaction mode is ‘(H – Bb) (H – Mj) (S – Bb) (L – Bb)’ (Figure 3a, right).

In the family ‘Restriction endonuclease EcoRV’ in a number of structures there is a contact of a loop with the DNA minor groove. For example in the structure 1BSU chain A has the interaction mode ‘(H – Bb) (S – Bb) (S – Mj) (L – Bb) (L – Mj)’ and the chain B has the interaction mode ‘(H – Bb) (S – Bb) (S – Mj) (L – Bb) (L – Mj) (L – Mn)’. It appears due to a minor variation of conformation of the Asn-70 side chain (Figure 3b). This means that this Asn residue is able to contact DNA but the chain A presents a ‘snapshot’ of the dynamical structure where occasionally the contact is absent. This example illustrates the reason why we use the union of types of interaction for different structures of one domain in determining the interaction class of a family.

One more factor that can affect interaction mode is the secondary structure determination. Short fragments of helices or beta-strands can be determined as unstructured segments due to minor variations in chain geometry. For example, in the family ‘Arc/Mnt-like phage repressors’ there is a structure 1BDT with four structures of the same domain. N-terminal parts of protein chains are detected by Stride as a part of an 3_10_-helix, for three chains (Figure 3c, right), and as a turn, for the chain A (Figure 3C, left). Thus for 1BDT, chain A, the interaction mode is ‘(H – Bb) (S – Bb) (S – Mj) (L – Bb) (L – Mj)’ and for 1BDT, chain B it is ‘(H – Bb) (H – Mj) (S – Bb) (S – Mj) (L – Bb)’.

As two last examples show, variations resulting in different interaction mode can be found even in subunits of one PDB entry.

### Problems in classification of families

The result of classification sometimes depends on quality of structures, especially if a protein domain is presented by a single structure of complex with DNA. Probably for a number of families the interaction class will be extended by additional types of contacts, when new (better) structures of the presented proteins appear. In a new structure a contact type can be detected that was occasionally not detected in previous structures. In this case the newly detected contact type will be added to the interaction class of the family. In the Table 1 such families are marked by the footnote “^a^".

A typical relationship between the interaction class of a family and interaction modes of members of the family can be illustrated with the example of the family ‘GalR/LacI-like bacterial regulator’, which contains 35 structures of three different proteins. Among these 35 structures seven interaction modes are observed, namely


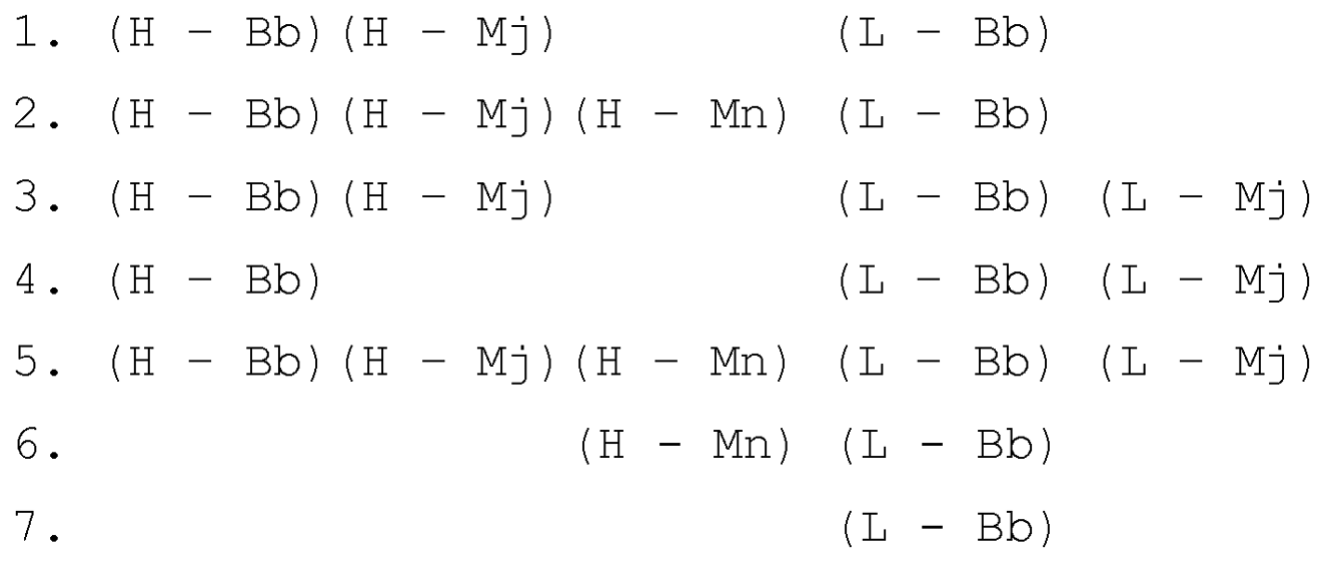


This diversity is due to absence of C-terminal helix (which interacts with the DNA minor groove) in some structures and also to crystallization artifacts of the complexes. Because each of the five contact types is present in structures of each of three proteins, the interaction class of the family is ‘(H – Bb) (H – Mj) (H – Mn) (L – Bb) (L – Mj)’.

For a number of families there are no contact types common for the entire family (the ‘miscellaneous’ interaction class). This situation can be observed in one of two cases.

1. Relative arrangement of DNA and protein is different in different structures while the family cannot be divided into well-defined subfamilies basing on the disposition. An example is the family ‘Classic zinc finger, C2H2’, where the following contact types are observed: (H – Bb), (H –Mj), (S – Bb), (L – Bb), (L – Mj), and none of these contact types is common for the entire family.
2. Relative arrangement of DNA and protein is common but DNA–protein interactions are made by amino acid residues in different positions. Thus in the family ‘C-terminal, Zn-finger domain of MutM-like DNA repair proteins’ the same beta-hairpin interacts *via* a beta-strand in one structures and *via* a turn in other structures, therefore interaction modes for different domains are (S – Bb) or (L – Bb) and the interaction class for the family is ‘miscellaneous’.

An X-ray structure represents a crystallized complex, thus it does not reflect possible dynamic contact and can contain artifacts. Therefore an analysis of DNA–protein contacts based on a single structure can be incomplete or even erroneous. Comparing related structures one can obtain an additional information on potential contacts. The proposed classification of DNA-recognizing domains allows selecting conserved contacts and determining potential artifacts. The algorithmic approach to the classification allows keeping it up-to-date despite of appearance of new structural information.

### DNA shape

The ‘shape readout’ recognition of DNA by proteins (see, e.g. (20,21)) can significantly contribute to the specificity of a DNA-recognizing protein. Often this shape readout is performed by dimers or even larger oligomers of DNA-recognizing proteins, and this effect is less evident at the level of single protein domains. For example, in complexes of transcription factors of the GalR/LacI family the DNA ‘kink’ occurs between the contact zones of two monomers of the protein. At the same time our approach at the moment is mainly domain-oriented, which restricts incorporation of DNA shape parameters into our classification. However even in contact zones of individual domains some regularities of the DNA shape can be observed.

To interpret the DNA shape parameters it should be noted that an ‘ideal’ DNA (e.g. generated by the program ‘fiber’ from the 3DNA package) has the following values of these parameters: zero Ax-bend, the minor groove 5.9 Å in width and 4.7 Å in depth, and the major groove 11.4 Å in width and 3.6–3.9 Å, depending on the base pairs, in depth. Calculation of the parameters in contact zones of protein domains shows that the minor groove is enlarged in width in the majority of cases. However there are three families, ‘Homeodomain’, ‘Interferon regulatory factor’, and ‘Recombinase DNA-binding domain’, where the average width of the minor groove in contact zones is close to that for the ‘ideal’ DNA. Note that there is a usual opinion that the minor groove is narrowed in the contact zone of homeodomains (see, e.g. (22)), but the direct statistical analysis does not confirm this, at least if to interpret ‘narrow minor groove’ as the minor groove less wide than of free DNA. On the other hand, the minor groove in contact zone with homeodomains is less wide than in contact zones with majority of other DNA-binding proteins. At the same time, for the family ‘Rel/Dordal transcription factors’ the minor groove is typically narrow (3.6–5.8 Å) even in comparison with the ‘ideal’ DNA. For three families, ‘TATA-box binding protein, C-terminal domain’, ‘HMG-box’ and ‘Prokaryotic DNA-bending protein’, the minor groove in contact zones is extremely enlarged (more than10 Å in width for all domains of these families).

The minor groove depth is highly correlated with the minor groove width: a narrow minor groove is also deep in most cases. On the contrary, the width of the major groove looks almost constant while its depth can be very different (from negative values to six or seven angstroms in some cases).

For many families of DNA-binding protein domains, the DNA in their contact zones is almost not bent. A significant DNA bend is obtained for ‘HMG-box’, ‘TATA-box binding protein, C-terminal domain’, ‘GalR/LacI-like bacterial regulator’, ‘SRF-like’ and ‘Prokaryotic DNA-bending proteins’ families. Note again that the observed values of Ax-bend (average values for these families are 2.4–5.1°) usually does not reflect the DNA bend in complexes with protein dimers.

## CONCLUSIONS

1. We updated NPIDB, adding a new classification of DNA–protein complexes and their families. These classifications take into account contacting structural elements of both DNA and protein. This allows users to navigate through NPIDB in less formal way, using not only titles of PDB files or protein or nucleic acid sequences, but also structural features of DNA–protein complexes.
2. We calculated a number of parameters of DNA shape in contact zones of DNA-binding domains and presented average values of these parameters for the families included in the classification.
3. We analyzed sources of diversity of DNA–protein interaction modes within one protein family. We show that the observed interaction mode is often influenced by artifacts of crystallization and secondary structure detection. We conclude that a classification of families can be more biologically meaningful than a classification of individual complexes.
4. Due to elaborated classification algorithms, our classifications can be easily updated after appearance of new structures of DNA–protein complexes.

## FUTURE DIRECTIONS

In the future, we plan to add the following features to NPIDB.

1. An automated procedure of addition of new complexes to the classification.
2. An analogous classification of Pfam protein domains.
3. An analogous classification of RNA-protein complexes, taking into account RNA secondary structures.

## AVAILABILITY

NPIDB is available online without registration at http://npidb.belozersky.msu.ru/. The new content is available via hyperlinks ‘Interaction classes’ and ‘Interaction modes’ in ‘Browse’ menu from each web page of the database.
